# THE IMPORTANCE OF MOSQUITO BEHAVIOURAL ADAPTATIONS TO MALARIA CONTROL IN AFRICA

**DOI:** 10.1111/evo.12063

**Published:** 2013-04

**Authors:** Michelle L Gatton, Nakul Chitnis, Thomas Churcher, Martin J Donnelly, Azra C Ghani, H Charles J Godfray, Fred Gould, Ian Hastings, John Marshall, Hilary Ranson, Mark Rowland, Jeff Shaman, Steve W Lindsay, T Meagher

**Affiliations:** 1 Fogarty International Center, National Institutes of HealthBethesda, Maryland 20892-2220; 3 Malaria Drug Resistance & Chemotherapy Laboratory, Queensland Institute of Medical ResearchBrisbane, Queensland 4006, Australia; 4 Epidemiology and Public Health, Swiss Tropical & Public Health InstituteBasel CH-4002, Switzerland; 5 University of BaselBasel CH-4003, Switzerland; 6 Infectious Disease Epidemiology, School of Public Health, Imperial College LondonLondon W2 IPG, UK; 7 Vector Group, Liverpool School of Tropical MedicineLiverpool, Merseyside L3 5QA, UK; 8 MRC Centre for Outbreak Analysis & Modelling, Department of Disease Epidemiology, Imperial College LondonLondon W2 1PG, UK; 9 Department of Zoology, Oxford UniversitySouth Parks Rd, Oxford OX1 3PS, UK; 10 Department of Entomology, North Carolina State UniversityRaleigh, North Carolina 27695; 11 Molecular & Biochemical Parasitology Group, Liverpool School of Tropical MedicineLiverpool, Merseyside L3 5QA, UK; 12 Department of Disease Control, London School of Hygiene & Tropical MedicineLondon WC1E 7HT, UK; 13 Mailman School of Public Health, Columbia UniversityNew York, New York 10032; 14 School of Biological and Biomedical Sciences, Durham UniversityDurham DH 3LE, UK

**Keywords:** *Anopheles*, indoor residual spraying, insecticidal nets, resistance

## Abstract

Over the past decade the use of long-lasting insecticidal nets (LLINs), in combination with improved drug therapies, indoor residual spraying (IRS), and better health infrastructure, has helped reduce malaria in many African countries for the first time in a generation. However, insecticide resistance in the vector is an evolving threat to these gains. We review emerging and historical data on behavioral resistance in response to LLINs and IRS. Overall the current literature suggests behavioral and species changes may be emerging, but the data are sparse and, at times unconvincing. However, preliminary modeling has demonstrated that behavioral resistance could have significant impacts on the effectiveness of malaria control. We propose seven recommendations to improve understanding of resistance in malaria vectors. Determining the public health impact of physiological and behavioral insecticide resistance is an urgent priority if we are to maintain the significant gains made in reducing malaria morbidity and mortality.

Long-lasting insecticidal nets (LLINs) and indoor residual spraying (IRS) are currently the key components of vector management strategies used for the control of malaria (Roll Back Malaria Partnership 2005). Over the past decade the use of LLINs, in combination with improved drug therapies, IRS, and better health infrastructure, has helped reduce malaria in many African countries for the first time in a generation (O'Meara et al. 2010; World Health Organization 2010). Malaria mortality has declined since 2000 by 25% globally and 33% in sub-Saharan Africa (World Health Organization 2011a). These remarkable successes have created unprecedented optimism about reaching the malaria reduction targets outlined in the Global Malaria Action Plan (Roll Back Malaria Partnership 2008) and, ultimately, for the local elimination of malaria; however, there is a growing threat to these gains in the form of insecticide resistance.

Evolution of resistance to the chemotherapeutic is a common outcome of effective (and ineffective) vector or parasite control programs. Although this is often viewed as a failure of the program, it is better regarded as an almost inevitable consequence because history has repeatedly shown that intensive interventions lead to the emergence of physiological (biochemical) resistance due to the high selective pressure exerted on the targeted population. The emergence of resistance in the vector has not only developed against all four classes of insecticide licensed to control adult mosquitoes for public health purposes (World Health Organization 1970; Ranson et al. 2011), but also in the malaria pathogen against the most widely used antimalarials, starting with chloroquine, the standard drug of treatment during the Global Malaria Eradication Campaign (GMEC) (Najera 1999). Indeed, resistance was one of the reasons cited for ending the GMEC in the late 1960s (Najera 1999). At that time resistance to DDT had developed in 14 anopheline species. The recent emergence of artemisinin drug resistance in South East Asia (World Health Organization 2011b; Phyo et al. 2012) makes clear that this is not simply a problem of the past, nor one confined to insecticides.

The finding of widespread physiological resistance to pyrethroids in *Anopheles gambiae*, the major vector of malaria in Africa (Ranson et al. 2011), is a major public health concern because pyrethroids are the only insecticides currently used for treating bed nets. Results from experimental hut studies in West Africa demonstrate a marked reduction in mosquito mortality in areas with high levels of physiological resistance (N'Guessan et al. 2007). Of course, in the absence of effective chemical control, intact bed nets still provide barrier protection against biting mosquitoes (Snow et al. 1988; Clarke et al. 2001); however, nets become worn and it is likely that torn or holed treated nets provide inadequate protection in areas where pyrethroid-resistant vectors are common (N'Guessan et al. 2007; Irish et al. 2008). This has recently been confirmed in studies at the same locations as the experimental hut trial (Asidi et al. 2012). In households in the areas where resistant mosquitoes were common there were high rates of blood feeding and freshly treated nets provided no protection once holed. In contrast, sleeping under a holed bed net in the location where susceptible mosquitoes were common decreased the odds of being bitten by 66% and the majority of mosquitoes were killed by the treatment.

At present there are 40 malaria-endemic countries reporting resistance to insecticides, most to pyrethroids (WHO Global Malaria Programme 2012). Multiple insecticide resistance is also common, with some regions having resistance to all four insecticide classes used in public health (Ranson et al. 2009). Although evidence is currently lacking that this level of resistance is impeding malaria control, most experts expect that current vector control efforts will soon be compromised unless strategies are implemented to manage the resistant vectors. It is estimated that more than half of the benefits gained from the current coverage of LLINs and IRS in Africa would be lost if pyrethroids lose their efficacy, resulting in approximately 120,000 additional deaths per year (WHO Global Malaria Programme 2012).

As a consequence of this growing threat, the World Health Organization Global Malaria Programme have published a Global Plan for Insecticide Resistance Management (GPIRM) (WHO Global Malaria Programme 2012). This strategy focuses solely on physiological resistance in malaria vectors. However, it is likely that behavioral resistance may also develop in response to insecticide exposure. Furthermore, how the behavior of physiologically resistant vectors might differ in comparison to their sensitive counterparts is very poorly known.

Behavioral resistance refers to any modification to mosquito behavior that facilitates avoidance or circumvention of insecticides. The contribution of behavioral changes in agricultural pests to insecticide/pesticide resistance has been long acknowledged (Sparks et al. 1989; Gould 2010), with theoretical studies providing valuable information to inform and improve management practices (Gould 1984; Castillo-Chavez et al. 1988). In comparison, determining if behavioral adaption in vectors may be of medical importance has lagged behind. Perhaps the best-documented behavioral change in malaria vectors, and the biggest concern, is the development of an early, outdoor feeding phenotype among anopheline populations in areas of extensive indoor insecticide use. These mosquitoes may circumvent LLIN and IRS control through preferential feeding and resting outside human homes and being active earlier in the evening before people have gone to sleep. In addition, there are a variety of other changes in vector behavior such as increased zoophagy that may evolve in response to intensive interventions. Part of the reason for the lack of information about behavioral resistance is that it is harder to investigate using relatively simple exposure assays, and far more difficult to monitor in field populations, compared to physiological resistance (Takken 2002; Ferguson et al. 2010).

Both physiological and behavioral resistance to insecticides may be determined by a limited number of major genes or be affected by a relatively large number of genes of small effect. The genetic basis of resistance affects the dynamics of spread as well as the ease with which molecular markers of resistance can be developed. Phenotypes caused by single gene mutations generally demonstrate an exponential increase in frequency, where much of the initial stage of spread occurs at very low, near-undetectable gene frequencies, prior to a period of rapid amplification to high frequencies. In contrast, phenotypes based on standing genetic variation in many genes typically have a different dynamic: their spread is generally described by an immediate and sustained change in their phenotypic distributions.

One cannot predict a priori which model will apply to a specific trait. Physiological resistance may occur through single mutations, for example, "knock-down resistance" (*kdr*) in the sodium channel protein targeted by pyrethroids, but may also arise through altered levels of detoxifying enzymes such as P450s and esterases, whose expression levels may well be modulated by variation in many genes, making it a quantitative genetic trait (Ranson et al. 2004; Wondji et al. 2009). Similarly, behavioral changes are often regarded as complex, quantitative genetic traits but there are instances of a single gene mutation in insects having large effects on behavior. For example, polymorphisms in the phosphoglucose isomerase (*pgi*) gene are associated with differences in butterfly dispersal rates as well as other phenotypic traits (Niitepold et al. 2009), while major mutations in some *Drosophila* circadian rhythm genes can affect their daily behavior cycles (reviewed in Sokolowski 2001). Another possible example is single gene mutations encoding physiological insecticide resistance, which also appear to change behavior through pleiotropic action that alters repellency (see later discussion). An important research gap is therefore a detailed understanding of the likely genetic basis of specific behavioral resistance traits, and how surveillance programs should be implemented to best monitor changes in these traits.

Here we review emerging and historical data on behavioral resistance in response to LLINs and IRS in an effort to understand better the biology underlying the field observations and highlight areas in need of further research. The data reviewed specifically focus on the *Anopheles* vectors of malaria, with an emphasis on sub-Saharan African species where much of the behavioral research has been conducted. The predominant species are *A. gambiae* sensu lato and *A. funestus*. *Anopheles gambiae* s.l. is a species complex consisting of several closely related sibling species including *A. gambiae* sensu stricto, *A. arabiensis*, *A. melas*, *A. merus*, *A. quadriannulatus* Species A, *A. quadriannulatus* Species B, and *A. bwambae*. There are a number of secondary vectors contributing to malaria transmission in sub-Saharan Africa, which we do not consider (Antonio-Nkondjio et al. 2006).

## Evidence for Impact of Indoor Insecticides on Mosquitoes

### VECTOR ABUNDANCE

The insecticides used for LLINs and IRS exert their effect on the vector population in three ways: toxic chemical action, spatial repellecy/deterrency, and contact irritancy (Box 1) (Smith and Webley 1968; Lines et al. 1987; Takken 2002). The relative importance of each of these in determining how an insecticide works is dependent not only on the chemical and concentration used, but also on the mosquito species (Dezulueta et al. 1963; Grieco et al. 2007; Chareonviriyaphap 2012) and the application methods (e.g., IRS vs. LLINs). The nontoxic chemical effects are highly relevant when assessing the impact of physiological resistance because it is the interaction between toxicity and behavior that determines the level of insecticide uptake and ultimately the probability that the insect dies.

**Box 1: Definitions**

*Anthrophagy*: species that feed on humans mainly.

*Contact irritant*: a chemical that stimulates mosquitoes to move away from the source after physical contact occurs.

*Endophagy*: species that have a preference to feed indoors.

*Endophily*: an inherent tendency to rest indoors after feeding (mosquitoes may feed indoors or outdoors).

*Exophagy*: species that have a preference to feed outdoors mainly.

*Exophily*: species that have a preference to rest outdoors mainly.

*Spatial repellent/deterrent*: a chemical that stimulates mosquitoes to move away from the source without the need for physical contact.

*Toxic chemical action*: knockdown or death of mosquitoes after physical contact with the chemical.

*Vectorial capacity*: the total number of infectious mosquito bites on humans that will arise from a single infected person on a single day.

*Zoophagy*: species that feed on animals mainly.

Data collected from experimental hut studies indicate that bed nets treated with pyrethroids and walls sprayed with DDT dramatically increase the rate at which African mosquitoes leave huts and reduce the number of blood-fed mosquitoes compared to untreated controls (Lines et al. 1987; Asidi et al. 2005; Chandre et al. 2010). This outcome suggests that these chemicals are contact irritants.

The evidence for spatial repellency, where mosquitoes are deterred from entering the house, is equivocal for treated nets, with some studies finding no reduction in the rate of entry (Kirby et al. 2008; Malima et al. 2008; Chandre et al. 2010; N'Guessan et al. 2010) while others reported significant decreases (Lines et al. 1987; Lindsay et al. 1991; Asidi et al. 2005). In less controlled settings, short-term use of treated nets did not appear to impact the number of *A. gambiae* s.l. entering houses (Mathenge et al. 2001), but instead acted as a contact irritant that increased exit rates, particularly of unfed mosquitoes, resulting in fewer mosquitoes resting indoors (Quinones et al. 1998; Mathenge et al. 2001; Akogbeto et al. 2011). Studies with IRS employing DDT suggested that the compound had some spatial repellence that may reduce mosquito entry into the house (Dezulueta et al. 1963; Smith and Webley 1968; Roberts et al. 2000). The level of deterrence reported for some experimental hut trials based on the number of mosquitoes collected indoors is sometimes confounded by the fact that mosquitoes entering the hut can leave by the same opening. This means that unless mosquito movements are carefully recorded through all openings, reductions in the rate of entry cannot be distinguished from unrecorded or higher rates of departure (Silver 2008).

Whatever the precise mechanism, the large-scale use of LLINs or IRS frequently results in a major reduction in the abundance of vectors, often referred to as the “mass community effect” (Hawley et al. 2003). This effect is the basis for the universal coverage advocated by Roll Back Malaria where the goal is that 80% of people at risk from malaria are protected by vector control methods, primarily LLINs and IRS (Roll Back Malaria Partnership 2005). Community surveys comparing villages with and without LLINs, or changes pre- and postintervention show decreased abundance of indoor resting mosquitoes (Mbogo et al. 1996; Lindblade et al. 2006; Bayoh et al. 2010), feeding mosquitoes (Trape et al. 2011), and larvae (Bayoh et al. 2010).

### MOSQUITO BEHAVIOR

There are a number of possible impacts that insecticide use indoors could have on mosquito behavior including changes in biting phenology and the frequency of endophagy. All anopheline vector species predominantly feed at night. One of the consequences of large-scale indoor insecticide use is the potential selection for vectors that feed on people earlier in the night while they are outdoors. Exophily was one of the reasons cited for why IRS (when used in isolation) failed to reduce malaria parasite rates substantially in the Garki project in northern Nigeria (Molineaux and Gramiccia 1980). Here large-scale use of IRS with propoxur, a carbamate insecticide, in an area of high transmission (entomological inoculation rate [EIR] = 18–145 sporozoite-positive bites per person each year) resulted in a 90% reduction in vectorial capacity, but only reduced the parasite prevalence for *Plasmodium falciparum* by 25% (the major vector was *A. gambiae* s.l.).

**Figure 1:**
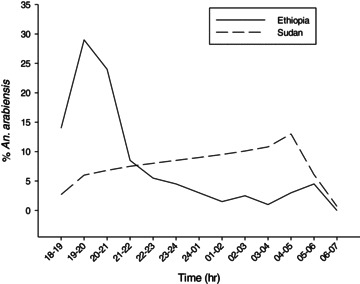
Distribution of biting times for *Anopheles arabiensis* after 40 years of DDT IRS in northern Ethiopia (Yohannes et al. 2005) and after 8 years of DDT IRS in northern Sudan (Dukeen and Omer 1986).

**Figure 2:**
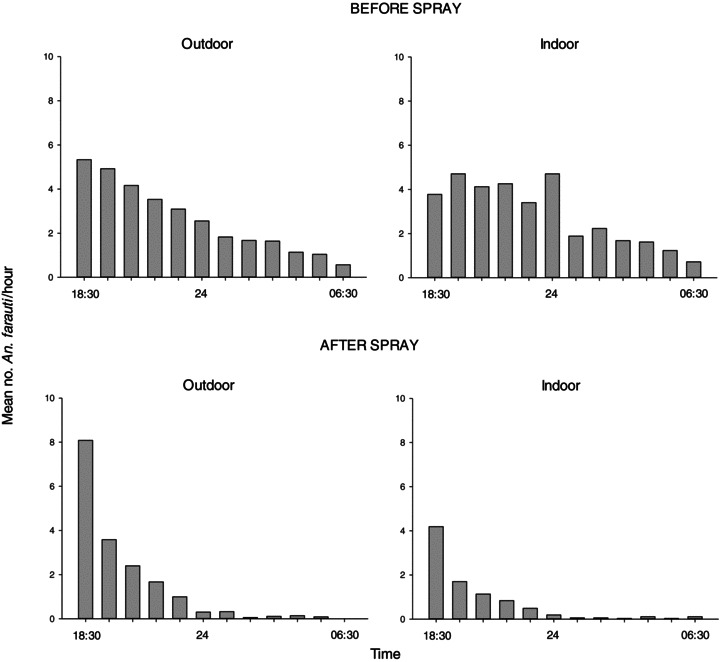
Distribution of biting times for *Anopheles farauti* before and after DDT spraying in the Solomon Islands (Taylor 1975).

*Anopheles arabiensis* populations show a wide range of peak biting times at different sites (Mattingly 1949; Dukeen and Omer 1986; Braack et al. 1994; Fontenille et al. 1997b; Lemasson et al. 1997; Yohannes et al. 2005; Yohannes and Boelee 2012), with some of this variation being explained by season (Tirados et al. 2006). One possible explanation for the remaining variation is that peak biting times may reflect the historical use of insecticides. Most interventions against this vector have involved IRS, which kills endophilic but not exophilic mosquitoes. Exophilic mosquitoes that are strongly anthropophagic need to feed early in the evening when humans are readily available outdoors. DDT has been used for IRS in Ethiopia for the last 40 years and there is some evidence in this country for increase exophily (Fig. 1) (Biscoe et al. 2005; Yohannes et al. 2005; Tirados et al. 2006; Yohannes and Boelee 2012). A clearer example of selection for early feeding comes from extensive indoor spraying of DDT to control *A. farauti* in the Solomon Islands (Taylor 1975). Prior to IRS the peak biting time for *A. farauti* was early evening, declining gradually until early morning. After extensive IRS the biting activity in the late evening and early morning almost disappeared, with most occurring in the early evening (Fig. 2). Although these examples demonstrate clear shifts in biting behavior, this response to indoor spraying has not been found everywhere. For example, in northern Sudan where indoor DDT spraying had been used for the 8 years, an entomological survey found "standard" biting cycle persisted in *A. arabiensis* (Fig. 1) (Dukeen and Omer 1986).

There are also mixed reports of the evolution of behavior in response to bed net deployment. Increased exophagy by *A. gambiae* s.l. in response to LLIN use has been reported in Kenya (Mbogo et al. 1996) but not Tanzania (Russell et al. 2011), while *A. funestus* exophagy increased significantly in Tanzania (Russell et al. 2011). Inside houses, the proportion of *A. gambiae* s.l. and *A. funestus* feeding before 2200 h increased, though peak biting still occurred after midnight (Mbogo et al. 1996; Russell et al. 2011; Trape et al. 2011). In addition, the proportion of early feeding mosquitoes increased following the introduction of LLINs, yet the absolute number of mosquitoes feeding during this time was generally less. This suggests that the observations may have resulted from failure to control a smaller population of residual mosquitoes that continued to bite earlier in the night. It should also be noted that the changes reported for *A. gambiae* s.l. do not account for any potential shifts in species composition within the complex. That is, the apparent behavioral changes could simply reflect effective control of a later-feeding member of the complex so that a previously less abundant species that feeds earlier in the night becomes relatively more dominant. In spite of these inconsistencies, the limited data available highlight the importance of monitoring for these behavioral changes in a range of settings, using a robust experimental and/or observational approach.

The difficulty and expense of accurately measuring mosquito behavior in the field has also limited the documentation and understanding of behavioral resistance. In one study, Mbogo et al. (1996) reported a reduction in the overall human biting rate from 95 to 34 bites per night, and an increase in the proportion of mosquitoes feeding outdoors from 1.2% to 30.3% following the introduction of LLINs. In the control village the biting rate remained constant at about five bites per person per night over the intervention period, and the proportion feeding outdoors increased from 2.7% to 20.3%. These results are difficult to interpret and extrapolate as sampling preintervention was conducted in five intervention and four control zones, whereas postintervention sampling was only conducted in one intervention and one control village. Data collected by pyrethrum spray collections from all the villages in the study indicated significant variations between sites meaning the pre- and postintervention data may not be directly comparable.

Results presented by Russell et al. (2011) are equally difficult to interpret. Their study used data from three field collections in Tanzania conducted (1) prior to the introduction of LLINs (1997), (2) after 75% of the community used untreated nets (2004), and (3) after 47% of the population used LLINs (2009) (Russell et al. 2011). No estimate of the overall biting rate is presented. However, graphical data suggest there is little difference in the overall biting rate between 2004 and 2009 for *A. gambiae* s.l., whereas biting rates for *A. funestus* are higher in 2009 compared to 2004. Compelling data are presented for decreasing endophagy rates for *A. funestus* over the time series, but not *A. gambiae* s.l., and a decreasing proportion of mosquitoes attempting to feed between 2100 h and 0500 h in both species (Russell et al. 2011). Interestingly, the trend for decreasing night feeding was consistent across the sample period, even though the coverage of LLINs only significantly increased in the second part of the time series, between 2004 and 2009.

### CHANGES IN VECTOR DOMINANCE

In parts of Africa the massive scale-up of LLIN deployment is associated with an apparent shift in vector dominance from the highly endophilic *A. gambiae* s.s. to the more exophilic *A. arabiensis*. This is seen in western and southern Kenya (Lindblade et al. 2006; Bayoh et al. 2010; Mutuku et al. 2011; Zhou et al. 2011) and Tanzania (Russell et al. 2011), but not Senegal (Trape et al. 2011). Recent data from western Kenya show an unexplained resurgence in *A. gambiae* s.s. during 2010 (Zhou et al. 2011). Species composition changes are also reported in coastal Kenya with significantly less *A. gambiae* s.s and more *A. merus* in intervention compared to nonintervention areas (Mbogo et al. 1996). However, as no preintervention data are presented, it is not possible to infer whether this difference is a result of an insecticide-induced change in species composition, or a difference in the initial species composition at the study sites. The change in species composition of adult mosquitoes at some sites is mirrored in the corresponding larval populations (Mutuku et al. 2011).

Analysis of data collected at Lupiro village, Tanzania beginning in 2002 shows a significant reduction in the relative proportion of *A. gambiae* s.s. compared to *A. arabiensis* over time leading the authors to conclude that high LLIN usage has dramatically altered the mosquito populations (Russell et al. 2011). However, closer examination of the data sources reveals a number of potentially confounding factors. For instance, the studies use a variety of collection methods including CDC light traps, human landing catches, resting collections, and “Ifakara traps,” and some report data from indoor collections only whereas others represent both indoor and outdoor collections. Also the studies are conducted at different times of the year relative to the wet and dry season, a factor known to differentially affect the abundance, feeding, and resting behavior of the vectors (Wanji et al. 2003; Cano et al. 2004; Koenraadt et al. 2004; Mutuku et al. 2011; Reddy et al. 2011; Trape et al. 2011). Unfortunately, several studies did not report when the collection was conducted, and the dry season collections tended to be clustered later in the study period confounding the interpretation of LLIN impact. Large variations (range: 4–96%) in the proportion of *A. gambiae* s.s. between sample clusters were also reported in at least one of the studies included in the analysis (Killeen et al. 2007), demonstrating the extreme variability in the ratio of *A. gambiae* s.s. to *A. arabiensis* in the village.

The above example highlights the complexity of assessing the exact impact of LLINs due to the background variability in the vector populations. Indeed, a substantial decline in vector numbers in the Tanga region of Tanzania where vector control has not been used on a large scale was reported between 1998 and 2009 (Meyrowitsch et al. 2011), highlighting the variability of malaria vector abundance. Relative species composition also varies greatly over time in natural populations as demonstrated by the unexplained change in dominant species from *A. funestus* to *A. arabiensis* between 1992 and 1995 in Dielmo village Senegal (Fontenille et al. 1997a), and the strong seasonal relationship between the relative abundance of *A. gambiae* s.s. and *A. arabiensis* (Highton et al. 1979; Lindsay et al. 1991; Dia et al. 2005; Oyewole et al. 2007; Trape et al. 2011).

In one series of experimental hut trials *A gambiae* s.s. was controlled more readily by LLINs than *A. arabiensis* despite having similar sensitivity to pyrethroid (Kitau et al. 2012). It is postulated that this differential mortality may be attributable to the more zoophilic *A. arabiensis* being less persistent in its attempts to bite the human host through the net than the more anthropophilic *A. gambiae*. Whatever the reason, the differential mortality provides one explanation for the possible shift in species ratio from *A. gambiae* s.s to *A. arabiensis* in areas with high coverage of LLINs.

Whether the propensity for outdoor biting by individuals of a given species is increasing or there is merely a residual population of outdoor-biting vectors is debatable, but the consequences of this change are important. Outdoor biting is difficult to counter with available control methods. Larval source management, spatial repellents, transgenic mosquitoes, and attractive toxic sugar bait could be used or developed for malaria control, but are either difficult to scale-up in all locations or are tools that will need much more research before they can be successfully deployed. The development of these, as well as new tools targeting outdoor-feeding mosquitoes is an urgent priority.

## What are the Population Dynamic Consequences of the Continued Use of LLINs and IRS?

Changes in vector abundance and species dominance are linked to processes affecting mosquito population dynamics. The observed abundance of mosquitoes is determined by the interaction of density-independent and density-dependent processes affecting mosquito survival and fecundity. The nature and action of the density-dependent processes is particularly critical as it sets the mean abundance about which populations fluctuate. We still know relatively little about *A. gambiae* s.l. population dynamics but most vector biologists believe that the most important density-dependent process involves competition amongst mosquito larvae for food (Smith and McKenzie 2004). A few studies that have manipulated larval densities in seminatural breeding sites show mortality increases relatively linearly with density (Gimnig et al. 2002; White et al. 2011). Understanding the location of density dependence in the mosquito life cycle relative to where insecticides act, as well as the shape of the mortality-density function, is important as it determines the degree to which insecticide deaths are compensated for by reduced density-dependent mortality; that is, it determines the impact of insecticide on vector population density (Hancock and Godfray 2007).

Reductions in mosquito abundance can have two further effects on disease transmission mediated through density dependence. There is evidence that lower larval densities increase survival, increase adult size, and lower development rate. As Lyimo and Koella (Lyimo and Koella 1992) among others has pointed out, increased size may be particularly pertinent to disease transmission if larger individuals live longer and so are more likely to survive through the disease latent period. Longitudinal surveillance data of mosquito size during an LLIN or IRS intervention would address this question, as would more data about the relationship between larval density and adult size, and adult size and longevity, in the field. Second, we know that the larval habitats for different members of the *A. gambiae* complex differ but overlap (Service 1973; Schneider et al. 2000). We do not know if these differences reflect adaptations to different niches or if different taxa compete with one excluding the other. If the latter is the case, then reducing the number of one type of mosquito may lead to competitive release of another. If the two mosquito taxa have different degrees of exophily/endophily then the ratio of mosquitoes feeding indoors or outdoors may change through interspecific population-dynamic processes.

Finally, the evolution of resistance typically entails fitness costs to the mosquito, which are most likely to be manifest when the insect is stressed, in particular when it is subject to density-dependent mortality (Kraaijeveld and Godfray 1997). We do not know the extent to which this happens, or indeed if it happens at all, but it is quite likely that the demographic and genetic dynamics of vectors are closely intertwined.

## How Concerned Should We Be About the Future Effectiveness of LLINs and IRS?

The key to the prolonged future success of LLINs and IRS is to understand the biological mechanisms underlying the changes being observed in the field. One possibility is that insecticide interventions are selecting for a heritable trait, that is, vectors that are genetically programmed to feed early outdoors. In this situation the effectiveness of LLINs in reducing malaria infection rates will decrease over time as the susceptible, indoor-feeding vectors are removed from the population, leaving predominantly the early outdoor feeders. There is clear evidence for a genetic basis for behavioral differences as the two closely related species *A. gambiae* and *A. arabiensis* often broadly differ in their feeding preferences and propensity to rest indoors or outdoors. In contrast, there are limited data on the role of genetics in behavioral polymorphisms within a species. It has been reported that there is an association between the 2R inversion polymorphism on chromosome 2 and differential endophily and endophagy in *A. arabiensis* (Coluzzi et al. 1977), but also that the preference of individual mosquitoes for a given resting location (indoor vs. outdoor) is not consistent (Lines et al. 1986), and that *A. arabiensis* shows site fidelity (returning to location of feeding) rather than host fidelity (McCall et al. 2001). It has also been suggested that feeding preferences for *A. gambiae* are related to the abundance of potential host species; in environments where there are many people the proportion of human blood-fed mosquitoes was high, but decreased when cattle were abundant (White 1974). However, the sporozoite rates were similar for mosquitoes, which were human blood-fed and cattle blood-fed, suggesting that the feeding preferences of *A. gambiae* s.s. are plastic (White et al. 1972). This overall plasticity in behavior allows continued mosquito survival when host species vary in abundance.

An alternative hypothesis, and one which presents a more promising outlook for LLINs, is that early outdoor feeding is a consequence of unsuccessful feeding on the prior evening. In this scenario, mosquitoes retain their inherent feeding preferences (e.g., location, host, and time) and in ideal situations will feed according to these preferences. With widespread LLIN coverage mosquitoes may not successfully feed indoors, being thwarted by the net barrier or repelled by the insecticide from the dwelling. Some of these mosquitoes may succeed in their search for a blood meal elsewhere, whereas others rest outdoors until the following evening at which time they recommence their search. These vectors may initiate their search soon after dusk and feed opportunistically on any outdoor hosts they encounter en route to a more preferred indoor feeding location. Under this hypothesis LLINs will continue to be effective as populations of indoor feeding mosquitoes will be retained, albeit with declining abundance caused by direct killing or increased mortality associated with delayed and nonoptimal feeding conditions.

Direct evidence supporting such a thwarted feeding theory is limited, although a similar model has been proposed to explain changes in mosquito host-seeking activities after IRS of houses with DDT (Roberts et al. 2000). Under this hypothesis the proportion of mosquitoes feeding early in the evening should be correlated with the probability of obtaining a blood meal; the lower the probability of finding the preferred late-evening blood meal, the more mosquitoes that feed early in the evening. Searching for new hosts (either human or animal), feeding on nonpreferred hosts and finding less suitable resting sites are all likely to be associated with increased mortality due to foraging risk, thus increasing the indirect impact of LLINs on mosquito survival and disease transmission. Previous modeling results have indicated that endemic disease transmission is highly sensitive to changes in mosquito survival during searching or feeding (Saul 2003). It is becoming increasingly clear that interventions such as LLINs and IRS are associated with dramatic reductions in malaria, which often exceed that expected based on measured changes in mosquito abundance alone (Trape et al. 2011). Thus unobserved secondary impacts, other than direct mosquito killing through toxicity, are likely to be occurring.

A critical appraisal and understanding of the biology underlying field observations is urgently needed to address the questions surrounding the longer term prospects of current interventions and assess potential new interventions. We need to be cautious about inferring selection of new behavior patterns when mosquitoes show an inherent plasticity in feeding when frustrated in accessing their hosts. Overall the current literature suggests behavioral and species changes due to LLINs may be emerging, but the data are sparse and, at times unconvincing and liable to publication bias, highlighting the need for greater research effort in this area. Only when these issues are better resolved can the future impacts of LLINs be fully predicted.

Modeling studies provide an important way of investigating the impact that physiological and behavioral resistance could have on disease prevalence; however, such studies are currently limited by a lack of understanding of the biological processes affecting insecticide resistance, particularly behavioral resistance. Theoretical predictions of the impact of IRS demonstrated over 30 years ago that model output is highly sensitive to assumptions regarding the uniformity of mosquito exposure to the insecticide (Molineaux et al. 1979), but there has been little advance in understanding the baseline distribution of exposure and if (or how) this changes following insecticide exposure. Resolving these issues will lead to improved models and better information for policy and control programs. Public health officials would then be able to address the key questions of whether resistance will compromise the long-term effectiveness of LLINs and IRS and how best to combat the problem.

To demonstrate the potential of mathematical models for investigating behavioral resistance we have selected one behavioral parameter, exophagy, and investigated its influence on the effectiveness of LLINS and IRS using two different comprehensive malaria transmission models (Smith et al. 2008; Griffin et al. 2010; Chitnis et al. 2012; OpenMalaria 2012) (see Box 2). Importantly, both models reach the same conclusion; that the impact of increased exophagy on EIR could be significant and of a magnitude comparable to, or exceeding, physiological resistance. There were also large differences in the predicted impact of resistance, particularly behavioral resistance, depending on the model assumptions regarding the structure of the mosquito population, specifically whether there is one homogenously mixed population or distinct populations of indoor and outdoor feeding mosquitoes. This preliminary modeling work highlights the importance of understanding mosquito behavior.

**Box 2: Modeling insecticide resistance and its impact on a combined LLN–IRS intervention**

The potential impact of insecticide resistance, both behavioral and physiological, on malaria transmission was assessed using two independent mathematical models: Imperial (Griffin et al. 2010) and OpenMalaria (Smith et al. 2008; Chitnis et al. 2012; OpenMalaria 2012). Both models incorporated the full malaria transmission cycle by including the mosquito life cycle as well as the human disease component. Three independent simulations were conducted using each model; (1) baseline simulation where 80% of the population used LLINs, which were replaced every 3 years and pyrethroid IRS treatment was applied every year to 80% of houses (both distributed at random), (2) physiological resistance where the interventions were the same as the baseline simulation but the effects of the insecticide (both killing and repellency) were reduced by 70% (though the physical effects of the nets remained the same), and (3) behavioral resistance where the interventions were the same as the baseline simulation but exophagy of the vectors was increased so that 70% of bites take place outside, with all other parameters kept constant. The two models assumed extremes of mosquito biting behavior; the Imperial model assumed only one population of mosquitoes that sometimes bit outdoors and sometimes indoors, whereas the OpenMalaria model assumed two populations of mosquitoes that either always bit indoors or always outdoors (Molineaux et al. 1979). All simulations assumed a half-life of 3 months for pyrethroid effectiveness with an exponential decay. The models were parameterized for *An. gambiae* s.s. with no seasonality. All other baseline parameters were as previously reported for the individual models: Imperial (Griffin et al. 2010) and OpenMalaria (Briët et al. 2012).

All simulations were calibrated so that the average EIR was 100 infectious bites per person per year prior to the introduction of the LLINs and IRS interventions. To investigate the impact of insecticide resistance (rather than the spread), it was assumed that the physiological or behavioral resistance was present when the interventions were introduced.

**Figure 3:**
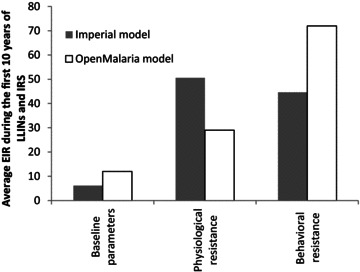
Predicted average EIR for the first 10 years of vector control (LLINs and IRS) using two mathematical models (Imperial and OpenMalaria) of malaria transmission.

Ten years after the start of the interventions the average EIR in both models decreased by approximately 90% to 6.1 and 12.0 infectious bites per person per year using the Imperial model and the OpenMalaria model, respectively (Fig. 3). As expected, the presence of physiological resistance reduced the impact of the interventions. Increased exophagy in the behavioral resistance simulations also decreased the effectiveness of the interventions; with the EIR predictions over 10 years after the start of the vector control being similar to, or higher than, those predicted for physiological resistance (Fig. 3). This suggests that the impacts of behavioral resistance could potentially be as severe, or even worse than, those of physiological resistance.

## Is It All Bad News?

Causing vectors to feed more often outdoors may actually represent new opportunities for control. Blood-feeding vectors can be captured in odor-baited traps (Okumu et al. 2010), killed by insecticide-treated cattle (Rowland et al. 2001), or after feeding on attractive toxic sugar bait (Muller et al. 2010), whereas gravid females might be targeted if we can develop effective oviposition traps (Harris et al. 2011). It is essential that new tools continue to be developed targeting outdoor-feeding mosquitoes, as their relative contribution to disease transmission will increase under successful LLINS and IRS campaigns. Behavioral changes favoring outdoor feeding and resting will also reduce vector exposure to insecticides inside the home, thereby reducing the selection pressure for physiological resistance.

The overall epidemiological effects of physiological insecticide resistance are not easy to estimate because the impact of an insecticide on individual mosquitoes is not only affected by genotype, but also their age and environment. Insecticide resistance is often strongest in young adults (Rowland and Hemingway 1987; Lines and Nassor 1991). The use of LLINS and IRS results in few mosquitoes surviving to be old enough to transmit malaria parasites so any (resistance) gene that increases survival during the first one or two gonotrophic cycles will have a major positive selective advantage. If as mosquitoes age the survival benefits of the gene decrease, many resistant mosquitoes may die before reaching the minimum infectious age. Hence malaria is still controllable, albeit to a lesser extent than in a purely sensitive mosquito population.

A side effect of physiological resistance is often a reduction in the behavioral responsiveness to the insecticide (Rowland 1990; Hodjati and Curtis 1997). For example, in one study, pyrethroid resistant mosquitoes show reduced irritability when in contact with the insecticide causing them to rest on the surface for longer periods than susceptible mosquitoes, thus increasing the dose of insecticide received (Hodjati and Curtis 1997). In most cases the effect of physiological resistance is unquantified and dependent on the mechanism of resistance (Rivero et al. 2010). There has also been a recent suggestion that insecticides may select for vectors that invest in short-term reproduction rather than longer term survival, resulting in a reduction in the number of older mosquitoes and a corresponding reduction in those able to transmit malaria parasites (Ferguson et al. 2012). For these reasons the overall consequences of accrued physiological and behavioral changes developed in response to the large-scale use of insecticides may not necessarily all be negative.

## The Way Forward

This review has highlighted a number of gaps in our knowledge of behavioral resistance in the vectors, which transmit malaria; conclusive evidence for the evolution of behavioral resistance has often been confounded by methodological issues. However, our preliminary modeling study has demonstrated that behavioral resistance could have a significant impact on the effectiveness of malaria control. As a result, we propose seven recommendations to improve understanding of both physiological and behavioral resistance in malaria vectors.

1. Develop robust methodologies for detecting specific types of behavioral resistance in the field.
2. Establish sentinel sites for long-term surveillance of physiological and behavioral resistance.
3. Improve understanding of the variability in behavior of individuals within a larger population of vectors (i.e., natural heterogeneity of population).
4. Report absolute mosquito abundance for each species in field studies, rather than reporting only proportional changes.
5. Determine whether apparent cases of behavioral resistance are due to heritable traits, and if so, develop diagnostic tests or identify a measured phenotype.
6. Better understand how physiological resistance may affect behavior, and consequently vectorial capacity.
7. Improve understanding of the behavior of male mosquitoes relative to exposure to insecticides via IRS and LLINS.

Determining the public health impact of both behavioral and physiological insecticide resistance is an urgent priority if we are to maintain the significant gains that have been made in reducing malaria morbidity and mortality over the past decade. Although there is still much research needed to understand better the spectrum of changes induced by intensive insecticide use, two points are paramount for future policy discussions. First, it must be remembered that interventions such as LLINs will provide some level of personal protection by presenting a physical barrier between sleeping hosts and mosquitoes, irrespective of the level of resistance, provided they remain in good condition. Therefore the development of insecticide resistance should never be a justification for removing or reducing the distribution of LLINs; rather, additional or modified interventions should be considered. Second, behavioral resistance cannot generally be addressed by simply changing insecticides. Instead, novel interventions exploiting new behavioral patterns are required. It is not unreasonable to recommend that interventions targeting outdoor feeding mosquitoes be the mandatory second phase of all intervention programs given the probability that resistance will eventually develop. At the moment this second phase is lacking from most intervention programs but the time has come to correct this.
